# A practical method for assessing quantitative scanner accuracy with long-lived radionuclides: The ARTnet insert

**DOI:** 10.22038/AOJNMB.2023.71860.1503

**Published:** 2024

**Authors:** Dale L Bailey, Kathy P Willowson, Carl Muñoz-Ferrada

**Affiliations:** 1 Department of Nuclear Medicine, Royal North Shore Hospital, Sydney, Australia; 2 Faculty of Medicine & Health, University of Sydney, Sydney, Australia; 3 Institute of Medical Physics, University of Sydney, Sydney, Australia; 4 Globalsonics Institute for Medical Research, Sydney, Australia

**Keywords:** Site validation, Clinical Trial, PET, SPECT

## Abstract

**Objective(s):**

To address the problem of using large volumes of long-lived radionuclides in test phantoms to check calibration accuracy of PET and SPECT systems we have developed a test object which (a) contains less radioactivity, (b) has a low total volume, and (c) is easier to store than currently used phantoms, while still making use of readily-available “standardised” test objects.

**Methods:**

We have designed a hollow acrylic cylindrical insert compatible with the NEMA/IEC PET Body Image Quality (IQ) phantom used in NU 2 performance testing of PET systems. The insert measures 90 mm internal diameter and 70 mm internal height and so is sufficiently large to not be subject to partial volume effects in PET or SPECT imaging. The volume of the insert is approximately 500 mL. It has been designed as a replacement for the standard long cylindrical “lung insert” in the IQ phantom without needing to remove the fillable hollow spheres of the phantom. The insert been tested with ^18^F, ^68^Ga and ^124^I PET/CT and ^99m^Tc, ^131^I and ^177^Lu SPECT/CT on scanners that had previously been calibrated for these radionuclides.

**Results:**

The scanners were found to produce accurate image reconstructions in the insert with  5% of the true value without any confounding uncertainty from partial volume effects when compared to NEMA NU 2-2018 Phantom measurement.

**Conclusions:**

The “ARTnet Insert” is simple to use, inexpensive, compatible with current phantoms and is suitable for both PET and SPECT systems. It does not suffer from significant partial volume losses permitting its use even with the poor spatial resolution of high-energy imaging with ^131^I SPECT. Furthermore, it uses less radioactivity in a smaller volume than would be required to fill the entire phantom as is usually done. Long-term storage is practical while allowing radioactive decay of the insert contents.

## Introduction

 Over the past 10-20 years nuclear medicine tomographic imaging has seen a greater emphasis on image quantification, that is, calibration of the imaging systems to produce reconstructed image values that correspond to an in vivo radioactivity concentration (Bq/cc). Quantitative PET and SPECT systems are now widely available (1). The past decade has also seen a large increase in the number of clinical trials involving imaging in nuclear medicine. 

 Much of this has been driven by the intro-duction of new theranostic agents and the need to generate high level evidence of their clinical efficacy as well as biodistribution and individualised dosimetry. The trials often involve quantitative imaging and therefore the site validation and initiation process usually requires in situ testing of the imaging equipment which will be used in the trial. For nuclear medicine this will include the dose calibrator, the gamma camera/SPECT (or SPECT/CT) system and/or the PET scanner (either PET/CT or PET/MRI) (2). Timing accuracy (time of day) is also important to ensure, particularly for short-lived radionuclides and the synchronisation of clocks or the use of a Master/Slave clock system is necessary. The need for accurate image reconstruction requires methods to calibrate and regularly verify the accuracy of the scanners.

 In response to these developments, in 2014 the nuclear medicine community in Australia and New Zealand created an imaging trials group called the Australasian Radio-pharmaceutical Trials Network, or “ARTnet”, to help facilitate multi-centre national trials in nuclear medicine (3). ARTnet has developed site validation protocols for quantitative imaging in both PET and SPECT based on a single phantom, the NEMA NU 2-2018 (PET) IEC/Image Quality (IQ) Phantom. 

 The analyses follow the measurements specified in the NEMA NU 2-2018 standard for performance testing of PET systems, which can also be applied in SPECT (4) with an additional analysis of the accuracy of quantitative image reconstruction using the Standardised Uptake Value (SUV). One suggested advantage of the IQ phantom is that it not a simple and highly symmetric right circular cylinder (or similar) but rather is “torso” shaped in cross-section and therefore may be more suitable for testing reconstruction algorithms incorporating attenuation and scatter correction. ARTnet has a number of NEMA IQ phantoms and coordinates their transportation around Australia and New Zealand in response to demand for site validation of trials or simply to verify quantitative accuracy for clinical use. In the five year period from 2016 ARTnet has validated over 100 PET cameras from 37 different scanners at 30 sites with both ^18^F and ^68^Ga (5). The ARTnet specification for quantitative image accuracy in PET is a reconstructed value within  5% of the true value and in SPECT it is within  10% of the true value.

 For clinical applications and imaging trials, short-lived radionuclides are mostly used as these have the desirable properties for use in man of short physical half-life and low radiation dose. Designing site validation procedures with these radionuclides does not create many issues in terms of handling of the radionuclides and storage of the test objects (or “phantoms”) after use as the radioactive contents decay naturally in a relatively short period of time. Examples of commonly used and tested radionuclides include ^18^F (t½=109.8 mins), ^64^Cu (12.7 hrs), ^68^Ga (68 mins), ^99m^Tc (6 hrs) and ^123^I (13.3 hrs). However, when evaluating a radionuclide such as one that would be used for therapy or one which is attached to a delivery moiety with a long biological clearance half-time such as a monoclonal antibody (mAb), longer-lived radionuclides are used. Examples of these include ^67^Ga (3.3 days), ^67^Cu (2.6 days), ^89^Zr (3.3 days), ^111^In (2.8 days), ^124^I (4.2 days), ^131^I (8 days) and ^177^Lu (6.7 days). If the radioactive contents of the test objects are unable to be disposed of into the waste system of the facility, which can be a restriction in some jurisdictions, they will need to be stored to allow for radioactive decay to reduce the radiation level before discharge. This can present a problem for storage as the phantom may be reasonably large and contain a large volume (up to  10 L) of radioactive fluid, especially for smaller facilities without suitable space to store long-lived radioactive sources, as well as rendering the phantom unable to be used for a significant amount of time.

 To address the issue of long-lived radionuclides “contaminating” test objects for significant periods of time we have produced an insert that is compatible with the NEMA IQ phantom, but which can be easily stored after use.

## Methods

***Insert Design***

 We have designed a small volume insert that can be introduced into a standard phantom as an alternative to filling the entire phantom with the long-lived radionuclide. The insert that we have developed, which we refer to as the “ARTnet Insert”, had the following design criteria:

*Compatible with NEMA/IEC IQ phantom;

* Suitable for use in both PET and SPECT;

*reusable;

*Not compromised by the partial volume effect;

*Low volume;

*Easily stored;

*Relatively inexpensive to produce with readily available materials.

**Figure 1:**
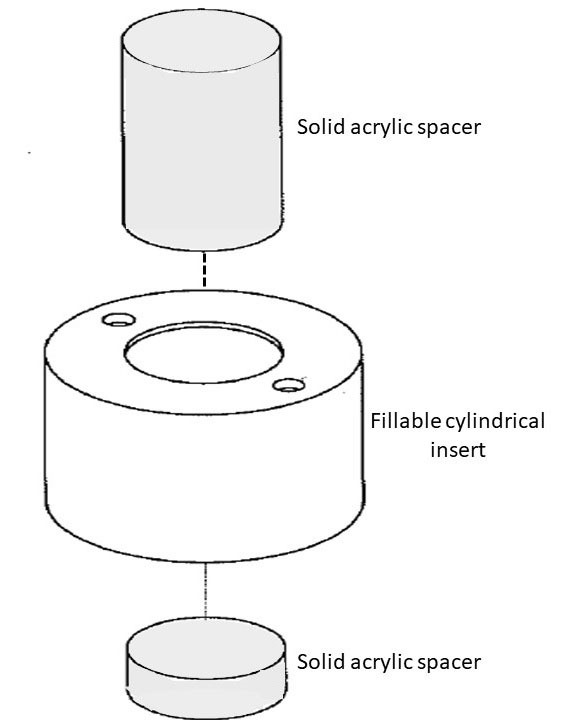
ARTnet Insert schematic drawing showing the hollow cylindrical insert and the solid spacers at either end which hold the insert in place in the NEMA phantom

**Figure 2:**
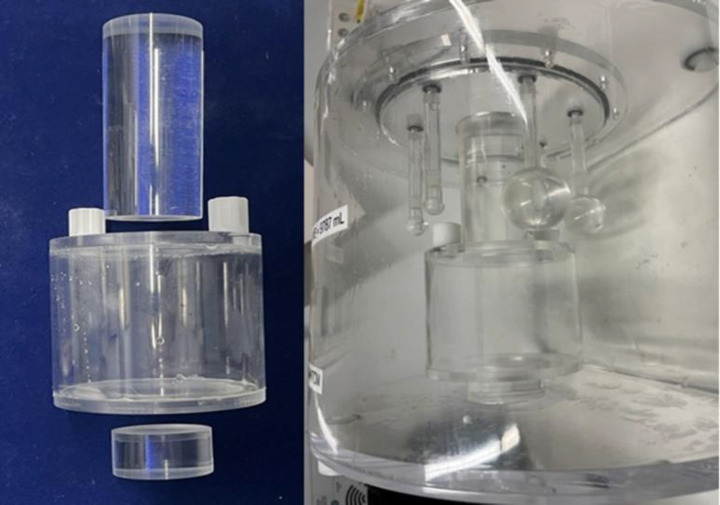
View of the empty insert and the solid acrylic spacers (**left**) and a side view of the insert positioned in the empty phantom where it has replaced the usual “lung insert” cylinder (**right**). Note that the fillable spheres are still in place in the phantom and do not need to be removed

 We decided to use a fillable circular acrylic cylinder (Figure 1). The insert is 100 mm in external diameter and 80 mm in external height. The walls of the insert are 5 mm thick and therefore the dimensions are 90 mm internal diameter and 70 mm height. It has circular recesses at each end into which solid acrylic cylindrical “spacers” are attached. The spacers are the same diameter as the normal cylindrical “lung insert” that is supplied with the NEMA/IEC IQ phantom which is usually filled with a mixture of polystyrene beads and water to simulate a low density area such as in the air-filled lungs. The ARTnet insert has two screw holes sealed with “O”-rings on one end for filling purposes. The insert with the spacers attached replaces the normal “lung insert” cylinder in the phantom. The spacers have been designed so that the hollow fillable spheres of varying sizes normally attached to the internal underside of one end of the phantom do not need to be removed to use the ARTnet insert (Figure 2). The inserts and spacers were produced locally from stock-supplied off-the-shelf materials to minimise cost and machined using a Computerised Numerical Control (CNC) router and leak tested prior to use. The volume of the insert is  500 mL.

***Data Acquisition and Reconstruction***

Testing was done for both SPECT/CT and PET/CT using similar approaches with different radionuclides on different days. Acquisitions were acquired with the ARTnet Insert in the IQ phantom (NEMA IEC PET Body Phantom (NU 2-2018), Data Spectrum Corporation, Durham, NC, USA (6)) with non-radioactive water surrounding the insert in the main compartment of the phantom and with radioactivity in the spheres. These measurements were compared with an acquisition at a different time of the IQ phantom with radioactivity in the background compartment as per the usual mode of use. 

 The SPECT/CT system used (Intevo 6, Siemens Healthineers, Hoffman Estates, IL, USA) has a 6-slice CT scanner and was fitted with appropriate collimators for each radionuclide – Low Energy High Resolution (LEHR) for ^99m^Tc, Medium Energy Low Penetration (MELP) for ^177^Lu and High Energy General Purpose (HEGP) for ^131^I. 

 The PET/CT used (Biograph mCT/64, Siemens Healthineers, Knoxville, TN, USA) has a 21.6 cm axial field of view and a 64-slice CT scanner incorporated. The radionuclides under test were mixed in 600 mL of tap water and added to the six spheres in the phantom followed by the ARTnet Insert. In this way the radioactivity concentration was the same in the spheres and the insert. The initial net amounts of radioactivity added to the 600 mL mixing volume were measured in a dose calibrator (Capintec, CRC 25-PET, Florham Park, NJ, USA) which had been validated against traceable standards for ^18^F, ^99m^Tc, ^131^I and ^177^Lu by our national standards laboratory (ANSTO, Lucas Heights, NSW). The amounts added to the diluting volume were 80 MBq of ^18^F, 64 MBq of ^68^Ga, 407 MBq of ^99m^Tc, 37 MBq of ^124^I, 106 MBq of ^131^I and 2160 MBq of ^177^Lu.

 SPECT data were acquired for 120 projections over 360° with continuous detector motion using the default energy window settings from the vendor and a Triple-Energy Window acquisition for scatter correction and quantitative imaging. All PET data were acquired for two overlapping bed positions centred on the phantom for 600 secs per bed position. The volume of the insert in which the radioactivity was diluted (600 mL) was converted to mass (600 g) and entered as the “Patient Weight” (in kg) in the acquisition-specific information to allow SUVs to be automatically calculated and displayed by the review and analysis software. The CT scans both used default “low dose” clinical settings; on the Intevo SPECT system the CT settings were tube voltage of 130 kV_p_ and the beam current was 25 mA while on the Biograph PET system the settings were 120 kV_p_ and 20 mA beam current. Both systems perform beam current modulation using Automated Exposure Control (AUC). Both systems produce reconstructions with a slice thickness of 3 mm.

 The accuracy measures from the acquisitions containing the ARTnet Insert were compared with our conventional NEMA IQ Phantom measurements with the radioactivity for all radionuclides diluted and mixed in 1270 mLs of water from which the hollow spheres in the phantom are filled while the remainder of the solution is added to the background compartment. This dilution procedure gives a spheres: background ratio of 8:1 for the IQ phantom.

 SPECT image reconstruction was performed on a dedicated nuclear medicine workstation (MIM Spectra, Cleveland, OH, USA) using energy-based scatter correction, CT-based attenuation correction and resolution recovery in an iterative OSEM reconstruction algorithm. This was done to minimise the number and variability of different reconstruction algorithms available from the vendors and standardise on a single algorithm and calibration approach where all free parameters in the reconstruction algorithm can be controlled. The PET reconstructions were performed on the Biograph mCT PET console system using an OSEM algorithm (1 iteration/21 subsets for ^124^I, 3 iterations/21 subsets for ^18^F and ^68^Ga) with Time-of-Flight localisation enabled and CT-based scatter and attenuation correction but without resolution recovery. The in-plane (x/y) pixel sizes in the final reconstructions were 3.9 mm (128 128 matrix) in SPECT and 4.1 mm (200 200 matrix) in PET. After initial calibration the systems are tested on a regular (monthly) basis as part of our routine departmental QA to maintain their quantitative accuracy varying the radionuclides each month. We also include a calibrated standard of  125 mL volume in a rectangular flask containing the same radionuclide as the subject has been administered in all quantitative acquisitions with clinical subjects and trial recruits as an internal calibration check (7) however this was not included in any of the phantom measurements reported in this work.

**Figure 3:**
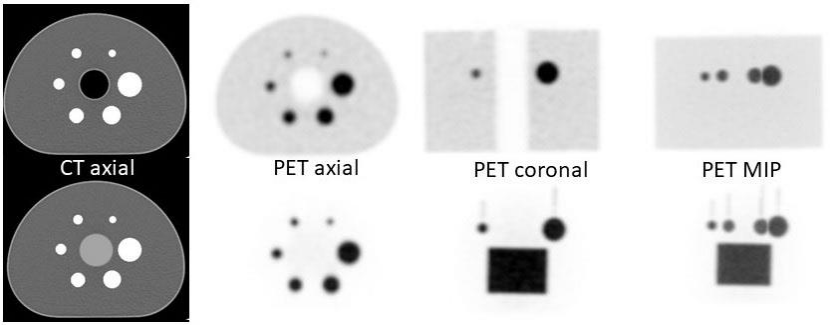
Comparative views of the NEMA IQ phantom (**top row**) and the ARTnet Insert in the phantom (**bottom row**) showing the segmented CT (**left**) and ^68^Ga PET images for axial, coronal and Maximum Intensity Projection (MIP) views are shown. There is no radioactivity in the background compartment of the phantom in the images with the ARTnet Insert. The axial views at the level of the central slices through the spheres does not show the insert however the solid acrylic spacer is visible in the centre of the phantom

 For the analysis of the ARTnet Insert acquisitions, a previously segmented CT scan of the phantom with each sphere and the insert defining a Volume of Interest (VOI) occupying the internal contents of each compartment was used (Figure 3). SUV_mean_ values were determined based on these VOIs after the SPECT or PET images of the phantom had been co-registered to the segmented CT phantom image. For the ARTnet Insert the SUV_mean_ is reported for a cylindrical VOI defined as 75% of the internal diameter of the cylinder in cross-section and covering the central 75% of the axial extent (similar to the concept of the Central Field of View (CFOV) for the NEMA measure of Gamma Camera uniformity). This was to avoid any partial volume or edge effects when using the entire internal volume of the insert. For the standard NEMA NU 2 acquisitions with radioactivity in the background compartment the SUV_mean_ and standard deviation was determined from the 60 prescribed background ROIs in the NEMA analysis.

## Results

***Image Accuracy***

**Figure 4:**
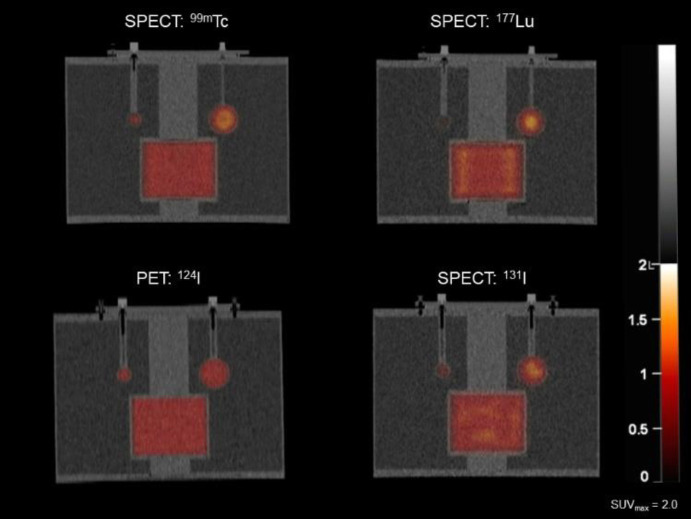
Coronal views of the ARTnet insert in the NEMA IQ phantom imaged with both SPECT and PET radionuclides as indicated. The smaller sphere shown is 18 mm and the larger is 37 mm in diameter. The radioactive solution is the same concentration in the insert as for the spheres. This is well demonstrated in the PET examples. In each situation the SUV_mean_ in the insert was within the specified limits of the expected value of 1.0. There is no radioactivity in the background compartment of the phantom in any of the images

 The analysis of the accuracy of the reconstructed images from the data acquisitions indicated are shown in Table 1. Example images are shown in Figure 4. 

 The results demonstrate that the insert approach displays a similar accuracy and closely matches the values derived from the NEMA NU 2-2018 analysis method which uses 60 pre-defined circular regions of interest (ROIs) across 5 separate transaxial sections in the background of the filled phantom.

**Table 1.** Mean SUVs in the NEMA IEC Phantom with and without the ARTnet Insert for a number of radionuclides are shown. The SUV_mean_ in the Whole Phantom was measured in the 60 Background ROIs specified in the NEMA NU 2-2018 analysis and the values for the ARTnet Insert were measured in a cylindrical VOI equal to 75% of the diameter of the insert and covering the central 75% of the axial extent of the insert as defined on a CT scan of the phantom with the insert in situ. The correct SUV in all cases is 1.0

**Radionuclide**	**Whole Phantom**	**ARTnet Insert**
	SUV_mean_	SUV_mean_
F-18	1.04±0.03	0.99±0.002
Ga-68	0.98±0.03	0.95±0.006
Tc-99m	1.01±0.05	0.98±0.02
I-124	1.04±0.03	1.02±0.01
I-131	1.02±0.03	0.98±0.02
Lu-177	0.97±0.05	0.99±0.03

## Discussion

 The requirement for the testing of nuclear medicine imaging equipment for clinical applications or for quantitative imaging trials is increasing. In general, the main characteristics of interest are overall image integrity, spatial resolution, signal-to-noise ratio and quantitative accuracy. Other measures such as Contrast Recovery Coefficient (CRC) and Background Variability (BV), as described in the NEMA NU 2-2018 specifications, are of secondary importance compared to quantitative image accuracy, recovery of true radioactivity concentration and spatial resolution. The insert that has been developed in this work addresses the accuracy issue in a practical way.

 ARTnet has chosen to perform all site validation testing using a single, commercially available phantom design for both PET and SPECT systems, the NEMA NU 2-2018 Image Quality phantom (3). This phantom is an irregular-shaped object and allows numerous parameters to be assessed and obtained including a number of extras in addition to those measured in the NEMA performance tests, such as Recovery Coefficients and SUV accuracy. The procedures are identical for assessing both PET and SPECT systems and therefore we designed the ARTnet Insert to be compatible with this test object to minimise costs and the need for multiple phantoms.

 The handling and storage of long-lived radionuclides can present a challenge, especially when combined with larger objects such as the NEMA/IEC IQ phantom of 10 L volume. When used with a radionuclide that cannot be disposed of in the facility’s normal liquid waste the phantom could require storage for up to 10 half-lives to reduce the concentration of the radionuclide to close to background levels ( 80 days for ^131^I). During this time the phantom effectively cannot be used for further testing. By combining a readily available phantom (NEMA/IEC IQ phantom) with a small insert we have minimised the need for additional test objects.

 The concept of using an insert in a pre-existing phantom arose during discussions with colleagues to explore standardising testing methodologies for clinical imaging trials in different regions of the world. The use of an insert in the NEMA IQ Phantom has been recently reported by Klarisvaart et al (8). They produced three contiguous cylindrical inserts which contained complex shapes that could be used to evaluate radiomic feature extraction in SPECT and PET. In contrast, our insert is a simple cylinder which only assesses accuracy over a restricted field of view. We envisage having a number of inserts available for each IQ phantom that we use so that, if necessary, after use with a long-lived radionuclide the insert can be retained by the site while its contents decay to background levels but the phantom can be sent on to the next testing site to be used with a different insert. Many facilities will not need to store the contents of the phantom and will be able to dispose of the radioactive solution soon after use, but others may need to keep the solution until it decays to background levels.

 Advantages of the ARTnet insert approach

 include its ease of storage, low volume of contaminated liquid contained, low amounts of radioactivity required to achieve the necessary concentration of radioactivity in solution and hence lower radiation exposure to staff handling the phantom as they are no longer handling the higher amounts of radioactivity needed for the larger background compartment of the phantom, and compatible with measurements in both SPECT and PET. When the same radioactivity solution is used to fill the spheres and the insert the images contain an “internal calibration” as the individual sphere concentrations, and hence recovery, can be compared with the large insert which is generally unaffected by spatial resolution effects.

 The insert still allows for the use of long-lived radionuclides in the fillable spheres to generate recovery coefficients. In the past, generating recovery coefficients from the spheres was usually done with radioactivity in the main compartment of the phantom such that there was a fixed ratio between the concentration in the spheres and the background in the main compartment; varying ratios such as 4:1, 8:1 and 10:1 have been used. However, observations and proposals in some recent presentations suggest that varying ratios may not be necessary. The first observation was that it is possible in SPECT to measure the recovery coefficients for a long-lived, medium energy radiotracer such as ^177^Lu by substituting a short-lived radiotracer (typically ^99m^Tc) but using the same collimators (e.g., MELP) that would be used for ^177^Lu imaging with the appropriate lower energy window (i.e., 140 keV ±10% for ^99m^Tc and 208±10% for ^177^Lu) (9). The short-lived radiotracer was found to give virtually identical results for Recovery Coefficients as would have been obtained by using the long-lived tracer in its energy window. While this requires further confirmation it would certainly provide a suitable workaround to having to fill the spheres with the long-lived radionuclide for SPECT. The second, more recent, suggestion was from the EARL standardisation committee of the EANM which proposed that recovery coefficients and spatial resolution for ^177^Lu could be assessed in the NEMA/IEC IQ phantom without any radioactivity in the main compartment and only in the fillable spheres (10). The phantom should contain non-radioactive (“cold”) water in the main background compartment. This means that only a very small amount and volume of long-lived radionuclide needs to be used to fill the spheres and therefore removing it after use from the hollow spheres and storing it until the contents have decayed should not be a problem. Either, or both, of these recent proposals will help to mitigate the problems associated with using long-lived radionuclides to measure recovery coefficients.

 Finally, the ARTnet Insert does not test the consistency of image reconstruction over the full axial extent of the test phantom in the way that the standard NEMA NU 2-2018 methodology does nor does it generate indices for background variability (BV) or contrast recovery coefficients (CRC). This is a limitation. It is not intended to replace the NEMA methodology for the full characterisation for an imaging system. However, it does provide a measure of the accuracy of calibration of the imaging system using a pragmatic approach. In addition, it is able to be used at the same time that Recovery Coefficients (RCs) from the fillable spheres can be measured with no crossover or interplay between the measurements as they are physically separated axially in the IQ Phantom.

## Conclusion

 A practical approach to testing quantitative image accuracy for long-lived radionuclides has been developed. The small insert which has been designed and constructed is simple but effective and relatively inexpensive. It replaces an existing insert in the NEMA/IEC IQ phantom and therefore avoids the need for new phantoms or other ways to reliably position it for testing using a readily available test phantom.

## Conflicts of interest

 DLB and KPW have no conflicts of interest to declare. Both are current or former members of the ARTnet Scientific Committee. CM-F is the owner and CEO of Globalsonics Institute for Medical Research Pty Ltd which has manufactured the ARTnet Insert and will make it available commercially.
